# The impact of a mobile app-based corporate sleep health improvement program on productivity: Validation through a randomized controlled trial

**DOI:** 10.1371/journal.pone.0287051

**Published:** 2023-10-05

**Authors:** Yuji Kawata, Sachiko Kuroda, Hideo Owan

**Affiliations:** 1 Graduate School of Economics, Waseda University, Tokyo, Japan; 2 Faculty of Education and Integrated Arts and Sciences, The Research Institute of Economy, Trade and Industry, Waseda University, Tokyo, Japan; 3 Faculty of Political Science and Economics, The Research Institute of Economy, Trade and Industry, Waseda University, Tokyo, Japan; Shahrood University of Medical Sciences, ISLAMIC REPUBLIC OF IRAN

## Abstract

Based on a randomized controlled trial applied to employees of a manufacturing company, this study examines the extent to which a corporate sleep program improves workers’ sleep health and productivity. In the three-month sleep improvement program, applicants were randomly divided into a treatment group and a control group, and the treatment group was provided with a noncontact sensing device to visualize their sleep. A smartphone app linked to the device notified them of their sleep data every morning and presented them with advice on behavioral changes to improve their sleep on a weekly basis. The results of the analysis revealed the following. First, even after controlling for factors that may cause sleep disturbances and nocturnal awakenings, such as increased workload and the number of days spent working from home during the measurement period, the treatment group showed improved sleep after the program compared to the control group. Second, the treatment group showed statistically significant improvement in presenteeism (productivity). The effect size on presenteeism through sleep improvement was similar regardless of the estimation method used (i.e., ANCOVA estimator of ATT and two 2SLS methods were performed). In particular, we confirmed that productivity was restored through sleep improvement for the participants who diligently engaged in the program. These results suggest that promoting sleep health using information technology can improve sleep deficiency and restore productivity.

## 1. Introduction

In this modern society, it is said that a significant proportion of people have problems with sleep, and sleep deprivation or sleep deficiency has become a global concern. For example, according to the Sleep Foundation [1], 44% of the respondents in the United States reported that they experience sleep problems almost every day. The *Comprehensive Survey of Living Conditions* (Ministry of Health, Labour and Welfare, 2019) also reports that approximately 30% of Japanese adults report that they do not get much or no rest from sleep (see also Kitamura et al. [2] and Ikeda et al. [3] for Japan, Stranges et al. [4] for Asia and Africa and van de Straat [5] for European countries). Many of these people may regard sleep deficiency as an inevitable part of life; however, if the lack of adequate sleep results in a loss of productivity, it is a major economic loss. The purpose of our paper is to investigate the extent to which the decline in productivity is caused by inadequate sleep and whether productivity can be improved if the quantity and quality of sleep are improved.

A number of previous sleep studies have shown that problems with sleep can cause serious impairments in daytime performance, such as motor vehicle accidents or work-related injuries (Leger et al. [6], Laugsand et al. [7]), worsen mental health (Soldatos et al. [8], Szklo-Coxe et al. [9]), and reduce quality of life (Guallar-Castillón et al. [10]). Regarding the relationship between sleep and work performance, Hafner et al. [11] reported that short sleepers of less than 6 hours per day had 2.4% and 1.5% lower productivity due to absenteeism and presenteeism, respectively, than those who sleep 7–9 hours per day (see also Loeppke et al. [12], Rosekind et al. [13], Swanson et al. [14], Hafner et al. [15], Ishibashi and Shimura [16] and Furuichi et al. [17]).

**Fig 1:**
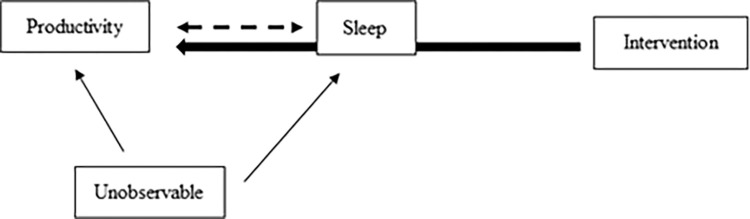
Causal relationship between sleep and productivity.

However, since most previous studies described above were based on cross-sectional data, identifying the causality between sleep and productivity is still a challenge. Just as there is an endogeneity problem in identifying the impact of sleep on health, the relationship between sleep and productivity is not clear (see, for example, Anderson and Bradley [18], Finan et al. [19], Johannessen and Sterud [20] and Cho and Chen [21]). In other words, rather than causality running from lack of sleep to productivity declines, there may be a reverse causality in which workers with low productivity are unable to complete their tasks on time and work longer hours, resulting in less sleep (see dotted arrow in Fig 1). As a result, a growing number of sleep studies have used randomized controlled trials (RCTs) to identify causal relationships (for example, Kaku et al. [22], Nishinoue et al. [23], Nakada et al. [24], Omeogu et al. [25], Okajima et al. [26] and Kjørstad et al. [27]; see also Robbins et al. [28] for a systematic review). As shown in Fig 1, if the intervention is randomly assigned, which means that the treatment subject receives is not correlated with the subject’s unobservable characteristics, we can examine the causal effect of sleep on productivity through the intervention (the bold arrow). Furthermore, to our knowledge, many previous intervention studies have mainly focused on patients who suffer from sleep disorders such as insomnia, and there have been only a few sleep intervention studies for general workers (see Burton et al. [29] and Redeker et al. [30]). While it is urgent to improve the sleep of patients suffering from insomnia, it is also true that there are many general workers with sleep deficiency (but not necessarily those with insomnia), even in the unwell stage, as mentioned above. We believe that more studies are needed on sleep improvement and the productivity of general workers using the RCT framework (Boubekri et al. [31]).

In this paper, by introducing the concept of *sleep health* proposed by Buysee [32], we investigate whether the improvement of sleep health improves work productivity through a randomized controlled trial. *Sleep health* is a concept that enables not only patients with sleep problems but also all people to achieve well-being and good performance through sleep. Specifically, by employing several dimensions of sleep health measures proposed by Buysse [32] that are related to health and performance outcomes, we conducted a 3-month RCT of sleep improvement for 215 employees of a large Japanese manufacturing company to determine whether employees who participated in a sleep improvement program gain sleep health compared to employees who do not participate and to what extent productivity improves when sleep health improves. In the sleep improvement program, the treatment group is provided with a noncontact sensing device that measures daily sleep data over a three-month period, notifies the person of the data every morning to visualize his or her sleep, and provides weekly advice on sleep improvement to encourage behavioral change through a smartphone app.

The contributions of this paper are as follows. First, we conducted an RCT in the general workforce to investigate the extent to which sleep health improves and work productivity improves as sleep health improves. As previous studies in sleep health have positioned presenteeism as a measure to capture productivity loss, we also consider presenteeism as productivity and use two measures in a self-response format: a composite indicator using 15 productivity-related questions in our original survey and the Work Limitations Questionnaire (WLQ) developed by Tufts University. Second, although there is a growing body of research demonstrating the impact of sleep health on physical and mental health (for example, Dong et al. [33], Lee and Lawson [34]), to the best of our knowledge, few studies have conducted RCTs on the relationship between improved sleep health and productivity. For sleep health, we selected six important aspects of sleep that may affect physical and mental wellness and performance: sleep duration, satisfaction/quality, alertness/sleepiness, efficiency of sleep, and timing and regularity as measures of sleep health based on Dong et al. [33] and Lee and Lawson [34]. Third, since we limit the subjects of our analysis to employees who work for the same company, there is no need to consider differences between employees working for different companies, such as management philosophy, corporate culture, corporate performance, and many other factors that may affect sleep. In addition to basic personal attributes to control for the heterogeneity among workers within the firm, we also control for a variety of factors that may affect sleep, including work-related factors (such as work tasks, the level of support from superiors and colleagues at the workplace, the number of days spent working from home, transfers and promotions) and major personal events (marriage, childbirth, and bereavement) that occurred during the three months of the program period. Fourth, we will focus on the treatment group and examine whether the degree of sleep improvement differs depending on the attributes and environment when RCTs are conducted.

## 2. Method

### 2–1. Randomized controlled trials

The randomized controlled trial of the sleep-improvement program analyzed in this paper was conducted among employees of a publicly traded Japanese manufacturing firm with more than 10,000 employees. The program was implemented over a six-month period from November 1, 2020, to the end of April 2021. Using a crossover RCT design, all subjects were randomly assigned to participate in one of the two periods (period 1: November to January, period 2: February to April). Of 215 participants who applied for the program, 157 were assigned to period 1 (treatment group) and 58 to period 2 (control group). The allocation to the two groups was unequal because of the firm’s decision to assign at least two-thirds of participants to the treatment group. Outcomes were assessed for the first period, with those assigned to period 2 serving as the control group.

Note that our datasets are proprietary and obtained in a legally restricted manner under confidentiality agreements with the firm and therefore cannot be made publicly available. The research team was provided with the anonymized data after the firm obtained written consent from all participants for the data to be used for academic research purposes. Specifically, this project was an opt-in, rather than mandatory, recruitment process. The firm clearly stated that the data collected would be analyzed within the firm to improve employees’ sleep and that once the internal analysis was completed, the anonymized data would be made available to Waseda University for academic research. Employees were also given the option to refuse to provide their data, but all participants in the project gave their written consent to provide their data. The authors notified the Ethics Review Committee of Waseda University that the program would be implemented by the firm and that the data would be provided for secondary use after the firm conducted an internal evaluation. The committee issued a decision indicating that no ethical review was required.

Participants were provided noncontact sensing devices from *NeuroSpace Co*., *Ltd*. during the term assigned. *NeuroSpace* is a Japanese venture company that develops sleep-sensing technology and simple evaluation algorithms and supports corporate health management through sleep improvement programs. The device, developed by *EarlySense*, an Israeli company, is placed under the bed mat and measures sleep status (bedtime, sleep latency, REM sleep, non-REM sleep, light sleep, number of awakenings, time of waking up, and actual time slept) based on heart rate and tossing and turning information. Note that Tal et al. [35] performed an epoch-by-epoch comparison of the *EarlySense* device and polysomnography in the sleep laboratory setting and reported that the system showed sleep detection sensitivity, specificity, and accuracy of 92.5%, 80.4%, and 90.5%, respectively. The daily sleep information obtained by the sleep measurement device is sent to a special app installed on the participant’s own smartphone, allowing the participant to check the daily sleep information (for details, see S1, S2 Appendices in S1 File). The participants received three elements of intervention during the three-month program: (1) they were able to check visualized information about their own sleep status every morning, (2) each week, they received a list of recommendations on how to improve their sleep and choose several items to achieve from the list, and (3) they were checked their compliance every night (advice was not just provided, but behavioral changes were encouraged by the app’s daily confirmation of the achievement status).

In addition, two seminars, which aimed to explain the app’s advice in detail and to provide knowledge about sleep hygiene, were held during the three-month program in period 1. However, participation in the seminar was voluntary, and to ensure fairness, the firm allowed not only the treatment group but also the control group to participate in the seminar. Since the data also identify the IDs of individuals who actually participated in the first and second seminars in both groups, the following analysis controls for seminar participation.

### 2–2. Data

For both the treatment and control groups, we used responses to self-administered surveys conducted before the start of the program (the end of October 2020; the baseline survey) and after implementation in period 1 (the end of January 2021; the follow-up survey).

**Fig 2:**
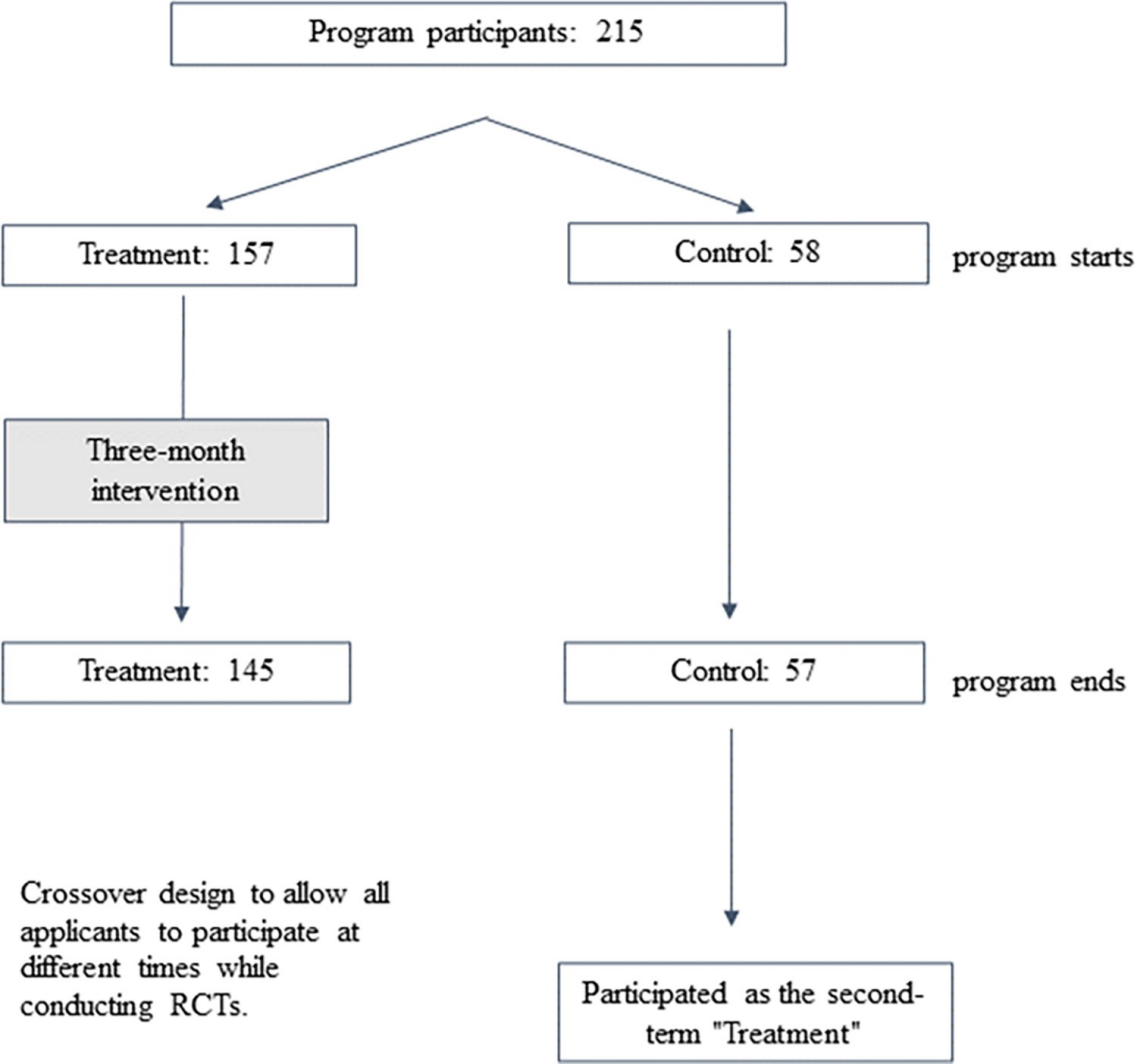
Program overview.

To measure the effect of the intervention, only the sample that responded to both the baseline and follow-up surveys was used in the following analysis. Because there were several participants who left the program or did not return the questionnaire, the final sample of participants who completed the follow-up survey included 145 observations in the treatment group and 57 observations in the control group (see Fig 2). The attrition rate was not large, at 7.6 and 1.7% for the treatment and control groups, respectively. There was no statistically significant difference in composition between the two groups at the beginning of the RCT. However, since several subjects dropped out after the RCT, we further checked possible differences between the two groups using the final sample of 202 participants. Table 1 compares the composition between the two groups. The rightmost column of Table 1 shows the results of the tests for differences between the two groups (t tests or chi-squared test) with p values, which indicate that there was no statistically significant difference between the two groups.

**Table 1:** Composition of the treatment and control groups.

	Treatment		Control		p-value
Age					
20s	37	(25.52)	14	(24.56)	0.42
30s	51	(35.17)	21	(36.84)	
40s	27	(18.62)	15	(26.32)	
over 50	30	(20.69)	7	(12.28)	
Sex					
male	97	(66.90)	32	(56.14)	0.15
female	48	(33.10)	25	(43.86)	
Family members living together					
single	49	(33.79)	17	(29.82)	0.42
infants and/or children	57	(39.31)	21	(36.84)	
spouse or partner	28	(19.31)	12	(21.05)	
others (sibilings, grandparents, roommates)	11	(7.59)	7	(12.28)	
Recent sleep satisfaction					
not very satisfied/not satisfied at all	98	(67.59)	35	(61.40)	0.15
very satisfied/satisfied	47	(32.41)	22	(38.60)	
Recent frequency of feeling sleepy					
almost never/once or twice a week	31	(21.38)	15	(26.32)	0.53
three or more times a week/almost every day	114	(78.62)	42	(73.68)	
Average hours worked per day in the last two weeks					
less than 10 hours	106	(73.10)	34	(59.65)	0.11
more than 10 hours	39	(26.90)	23	(40.35)	
Frequency of feeling depressed in the last 2 weeks					
almost never/sometimes	95	(65.52)	37	(64.91)	0.76
often/almost always	50	(34.48)	20	(35.09)	

#### 2-2-1. Sleep-related behaviors

The baseline and follow-up surveys asked essentially the same nine questions about sleep-related behaviors, which were used in the analysis to examine whether the program resulted in changes in participants’ behaviors that led to improved sleep. The specific questions are indicated in S1 Table in S1 File. Variables were created so that the greater the number was, the higher the frequency of each behavior.

#### 2-2-2. Sleep health measures

To test the extent to which RCTs improve sleep health, we used the following six sleep health scale items in accordance with Buysee [32], Dong et al. [33], and Lee and Lawson [34]: sleep satisfaction, daytime alertness/sleepiness, sleep efficiency, sleep duration, timing, and regularity. The specific questions used to measure these scale items are described in Table 2. Using them, we created two sleep health scores, *SH1* and *SH2*. *SH1* is a simple summation of dummy variables created from the six items with a 4-point scale dichotomized into 1 and 0 (see Table 2 for details). *SH2* is a composite index created using correspondence analysis applied to eleven 4-level category variables (except “timing”) related to sleep health (see also Table 2 for details). The index is created in such a way that the larger the value is, the better the sleep and is normalized with one corresponding to one standard deviation. *SH1* is similar to the scores used in Dong et al. [33] and Lee and Lawson [34] and therefore comparable to those of previous studies. *SH1*, however, may have problems accessing an appropriate duration of sleep since it treats short and long sleep durations equally. As pointed out by the results of several previous studies, the effects of long sleep duration are mixed (see, for example, Watson et al. [36]. Therefore, it is better to measure the sleep duration in a nonsymmetric form, and we believe that the correspondence analysis used to create *SH2* can capture the nonsymmetric effect.

**Table 2:** Summary of the six sleep health variables.

	Questionnaire	SH1	SH2
sleep satisfaction	"Please select your recent level of satisfaction with your own sleep."	Used as a dummy variable that takes 1 if answer is "very satisfied"/"satisfied" and 0 if "not very satisfied"/"not satisfied at all"	Used as a 4-level category variable: "very satisfied" 4, "satisfied" 3, "not very satisfied" 2, and "not satisfied at all" 1
daytime alertness	"Do you ever feel sleepy, such as yawning, drowsy, or tired within 4 hours of waking?"	Used as a dummy variable that takes 1 if answer is "almost never"/"once or twice a week" and 0 if "three or more times a week"/"almost every day"	Used as a 4-level category variable: "almost every day" 4, "three or more times a week" 3, "once or twice a week" 2, and "almost never" 1
	"Do you feel drowsy at work?"	–	
sleep efficiency	"Do you ever wake up more than an hour earlier than you intended to and then have trouble going back to sleep?”	Used as a dummy variable that takes 1 if answer is "almost never"/"once or twice a week" and 0 if "three or more times a week"/"almost every day"	
	"Do you have trouble sleeping through the night and wake up in the middle of the night for some reason?"	–	
	"When you wake up, do you feel your mind is foggy and you can’t get up right away even when the alarm goes off?"	–	
	"After getting into bed, I think a lot and repeatedly turning over in bed, and finally fall asleep after more than 30 minutes."	–	
	"I fall asleep so fast that I lose consciousness (within 5 minutes) when I get to bed."	–	
duration	"What time did you usually go to bed in the last two weeks?" "What time did you usually wake up in the last two weeks, ?"	Used as a dummy variable that takes 1 if "waking time-bedtime" is 6-8 hours and 0 if less than 6 hours / more than 8 hours .	Used as a 4-level category variable: "less than 5 hours" 1, "5-6 hours" 2, "6-8 hours" 3, and "more than 8 hours" 4.
timing	"In the last two weeks, what time did you usually go to bed?" "In the last two weeks, what time did you usually wake up?"	Used as a dummy variable that takes 1 if the midpoint is in the normal range of 2 AM to 4 AM and 0 if not	Used as a dummy variable that takes one if the midpoint is in the normal range of 2 AM to 4 AM and 0 if not
regularity	"Do you ever sleep longer than your workday on your days off?"	Used as a dummy variable that takes 1 if answer is "rarely"/"less than half" and 0 if "more than half"/ "every time"	Used as a 4-level category variable: "every time" 4, "more than half" 3, "less than half" 2, and "rarely" 1
total		Simply sum six dummy variables and convert to a score of 0-6	Convert all items into a composite index by correspondence analysis

#### 2-2-3. Productivity

We use two indices of productivity, *Productivity indices* 1 and 2, to examine to what extent work productivity improved as a result of improvement in sleep health through the sleep improvement program. To create the former, we use the responses to the 15 questions provided in the baseline and follow-up surveys (the questions are described in S1 Table in S1 File).

Respondents were asked to answer each of these questions on a 10-point Likert scale, with higher numbers indicating a greater frequency. Responses to these 15 items are transformed into a composite variable, *Productivity Index 1*, by correspondence analysis.

As the second productivity index, we use presenteeism data based on the *Work Limitations Questionnaire* (WLQ; see Lerner et al. [37, 38]) The data were provided by the firm, which conducts the WLQ survey of all employees every year in September and October. The WLQ has been translated into multiple languages, and the WLQ-J, which is a Japanese translation of the WLQ, was used in this study (for an explanation of the WLQ-J, see Ida et al. [39]). Since the survey was conducted just prior to the implementation of the sleep improvement program, the firm administered the WLQ survey to the program participants (both treatment and control groups) after period 1 (at the end of January 2021) to verify the effectiveness of the program.

As in *Productivity index 1*, the higher the value in *Productivity index 2* is, the higher the productivity. The WLQ has four subscales (time management, physical tasks, mental-interpersonal tasks, and outcome tasks) and a total scale that combines them.

The number of participants who responded to both baseline and follow-up WLQ surveys was 114 in the treatment group and 39 in the control group. Using these samples, we also conducted tests of the difference in composition as well as *Productivity Indices 1* and *2* between the two groups before the RCT and confirmed that there was no statistically significant difference.

#### 2-2-4. Control variables

Considering possible compounding factors that may affect both sleep and work productivity, we try to control for a variety of work and workplace conditions in the analysis below.

Table 3 lists the basic statistics for the primary data used in the analysis (see S1 Table and S1, S2 Appendices in S1 File for further details of other data used in the analysis).

**Table 3:** Basic statistics.

	Full sample			Treatment group			Control group		
	(1)	(2)	(3)	(4)	(5)	(6)	(8)	(9)	(10)
	sample sizes	mean or n	standard deviation or %	sample sizes	mean or n	standard deviation or %	sample sizes	mean or n	standard deviation or %
**Age**									
20s	404	102	25.2%	290	74	25.5%	114	28	24.6%
30s	404	144	35.6%	290	102	35.2%	114	42	36.8%
40s	404	84	20.8%	290	54	18.6%	114	30	26.3%
over 50	404	74	18.3%	290	60	20.7%	114	14	12.3%
**Sex**									
male	404	258	63.9%	290	194	66.9%	114	64	56.1%
female	404	146	36.1%	290	96	33.1%	114	50	43.9%
**Family members living together**									
single	404	132	32.7%	290	98	33.8%	114	34	29.8%
infants and/or children	404	156	38.6%	290	114	39.3%	114	42	36.8%
spouse or partner	404	80	19.8%	290	56	19.3%	114	24	21.1%
others (siblings, grandparents, roommates)	404	36	8.9%	290	22	7.6%	114	14	12.3%
**Sleep health scores**									
SH1	402	4.32	1.19	288	4.38	1.20	114	4.18	1.13
SH2	402	-0.00	1.00	288	0.05	0.99	114	-0.13	1.04
**Sleep Satisfaction**									
the level of satisfaction with recent sleep	404	2.37	0.66	290	2.40	0.66	114	2.29	0.65
**Daytime alertness**									
feel sleepy, such as yawning, drowsy, or tired within 4 hours of waking	404	1.78	0.92	290	1.76	0.94	114	1.82	0.86
feel drowsy at work	404	1.87	0.87	290	1.82	0.84	114	2.00	0.93
**Sleep efficiency**									
wake up more than an hour early and have trouble going back to sleep	404	1.41	0.76	290	1.41	0.80	114	1.39	0.65
have trouble sleeping through night and wake up in the middle of the night	404	1.85	1.05	290	1.92	1.08	114	1.68	0.97
feel foggy and can’t get up right away even when the alarm goes off	404	2.22	1.15	290	2.10	1.11	114	2.52	1.21
think a lot, repeatedly turning over in bed, takes more than 30 min to fall asleep	404	1.97	1.03	290	2.01	1.04	114	1.84	1.00
fall asleep so fast that I lose consciousness within 5 minutes	404	2.27	1.14	290	2.22	1.12	114	2.41	1.18
**Sleep duration**									
< 5 hours	402	8	2.0%	288	7	2.4%	114	1	0.9%
5 hours < = x < 6 hours	402	28	7.0%	288	19	6.6%	114	9	7.9%
6 hours < = x < = 8 hours	402	327	81.3%	288	234	81.2%	114	93	81.6%
> 8 hours	402	39	9.7%	288	28	9.7%	114	11	9.6%
**Timing**									
if midpoint is earlier than 2 AM or later than 4 AM	402	90	22.4%	288	60	20.8%	114	30	26.3%
if midpoint is in the normal range of 2 AM to 4AM	402	312	77.6%	288	228	79.2%	114	84	73.7%
**Regularity**									
sleep longer than workday on days off	404	2.33	1.16	290	2.28	1.14	114	2.46	1.23

## 3. Estimation strategy

### 3–1. Analysis of covariance (ANCOVA)

According to McKenzie [40], the following ANCOVA estimator of Eq 1 has more power to test for RCTs than a difference-in-differences analysis. Based on McKenzie [40], we employ the ANCOVA estimator to measure the effect of the RCT.

$${\hat{\gamma }}_{ancova}=({\bar{Y}}_{POST}^{T}-{\bar{Y}}_{POST}^{C})-\hat{\theta }({\bar{Y}}_{PRE}^{T}-{\bar{Y}}_{PRE}^{C}).$$ (1)

where ${\bar{Y}}_{POST}^{T}=\frac{1}{n_{T}}\sum_{i\in T}Y_{i1},\;{\bar{Y}}_{POST}^{C}=\frac{1}{n_{C}}\sum_{i\in C}Y_{i1},\;{\bar{Y}}_{PRE}^{T}=\frac{1}{n_{T}}\sum_{i\in T}Y_{i0}$, and ${\bar{Y}}_{PRE}^{C}=\frac{1}{n_{C}}\sum_{i\in C}Y_{i0}.\;Y_{i,t}$ are the outcome measures for worker *i* from the baseline (t = 0) and follow-up (t = 1) surveys, and *n*_*T*_ and *n*_*C*_ are the number of workers in the treatment group and in the control group, respectively.

Eq 1 can be estimated by running an OLS regression of the following equation:

$$Y_{i1}=\gamma Treatment_{i}+\theta Y_{i0}+\epsilon_{i},$$ (2)

where ${\hat{\gamma }}_{ancova}$ and $\hat{\theta }$ of Eq 1 are the estimates of *γ* and *θ* of Eq (2), *Treatment*_*i*_ is a binary variable that takes the value of one if individual *i* is assigned to the treatment group and zero if individual *i is* assigned to the control group, and ϵ_*i*_ denotes the error term. The outcome measures include the behavioral changes related to sleep, the sleep health indicators *SH1* and *SH2*, six subcategories of sleep health indicators, and two presenteeism measures (*Productivity index 1 and 2*). To further improve the power, we control for individual attributes and major events in both work and personal life during the program period described in 2.2.4 by including covariates *X*_*i*_ in Eq 3 below.


$$Y_{i1}=\gamma Treatment_{i1}+\theta Y_{i0}+\beta X_{i}+\epsilon_{i}.$$ (3)

### 3–2. Two-stage least squares (2SLS)

The treatment effect (γ) of the sleep improvement program calculated in Eq 3 is the average effect for all participants (ATT). We might interpret it as the intent-to-treat (ITT) effect because there may be a certain number of participants who did not use the app or follow the advice at all and thus did not change their behavior. In fact, in the follow-up survey, there were participants who stated that the program was too troublesome, and they did not take it very seriously (27.8%). Some said that they got bored in the middle of the program and did not try as hard to comply in the latter half (8.3%) or that they tried to comply, but it depended on their mood (8.3%). On the other hand, 9.0% of the participants answered that they actively tried their best, and 46.5% were able to try to some extent, indicating that approximately half of the participants actively participated in the sleep improvement program to some degree.

Since the estimation of Eq 3 represents the average effect of the assigned intervention, there could be a large variation in the effects of intervention on sleep and productivity if there is a certain proportion of “noncompliers.” Therefore, in what follows, we use 2SLS to estimate the Local Average Treatment Effect (LATE) of diligent participation. Specifically, we used the dummy variable *Z*_*i*_, which represented individual *i*’s degree of diligence toward the program (the responses to “I was able to actively try my best in the program” and “I was able to try in the program to some extent” were set to 1 and 0 otherwise) as the endogenous right-hand side variable. We then estimated Eq 4 as the first-stage estimation using *Treatment*_*i*_ as the instrument along with covariates *X*_*i*_. Eq 5 corresponds to the second-stage estimation, where *Y*_*it*_ is the outcome measure.


$$Z_{i}=\gamma_{1}Treatment_{i1}+\theta_{1}Y_{i0}+\beta_{1}X_{i}+\eta_{i},$$ (4)

$$Y_{i1}=\gamma_{2}Z_{i}+\theta_{2}Y_{i0}+\beta_{2}X_{i}+\epsilon_{i}.$$ (5)

In addition, note that only 58 people (approximately 40%) in the treatment group had higher posttreatment sleep satisfaction than preintervention, while 8 people (14%) in the control group reported improved sleep satisfaction. Since less than half of the treatment group also experienced improved sleep, it is necessary to determine the extent to which the intervention improved sleep and the extent to which productivity improved through that pathway. Even if the interventions did improve productivity, we do not know if all the improvement was realized through improved sleep health. For example, people may have become more conscious of their health and reviewed their dietary and exercise habits during the program, which in turn might have increased productivity through improved physical condition. It is also possible that the subjects’ assignment to the treatment group increased their loyalty to the company and raised their productivity as a result of working more diligently.

If the ATT analysis includes effects through pathways other than sleep improvement, it is not possible to elucidate the extent to which sleep improvement contributed to the improvement in productivity. Therefore, we estimated the causal effect of sleep improvement by 2SLS as follows. Using the sleep health scores *SH2* as the endogenous right-hand side variable, we estimated Eq 5 as a first-stage estimation using *Treatment*_*i*_ as the instrument along with covariates *X*_*i*_. Note that *S*_*i*1_ and *S*_*i*0_ are the posttreatment and pretreatment sleep health scores *SH2*. Eq 6 corresponds to the second-stage estimation, where *Y*_*it*_ is the outcome measure.


$$S_{i1}=\gamma_{1}Treatment_{i1}+\theta_{11}S_{i0}+\theta_{21}Y_{i0}+\beta_{1}X_{i}+\rho_{i},$$ (6)

$$Y_{i1}=\gamma_{2}S_{i1}+\theta_{12}S_{i0}+\theta_{22}Y_{i0}+\beta_{2}X_{i}+\epsilon_{i}.$$ (7)

In what follows, we estimated the impact of the intervention on sleep health scores *SH1* and *SH2* and then estimated the impact on two productivity indices by ANCOVA and 2SLS through diligent participation in the program and sleep improvement.

## 4. Estimation results

### 4–1. Changes before and after the program

**Fig 3:**
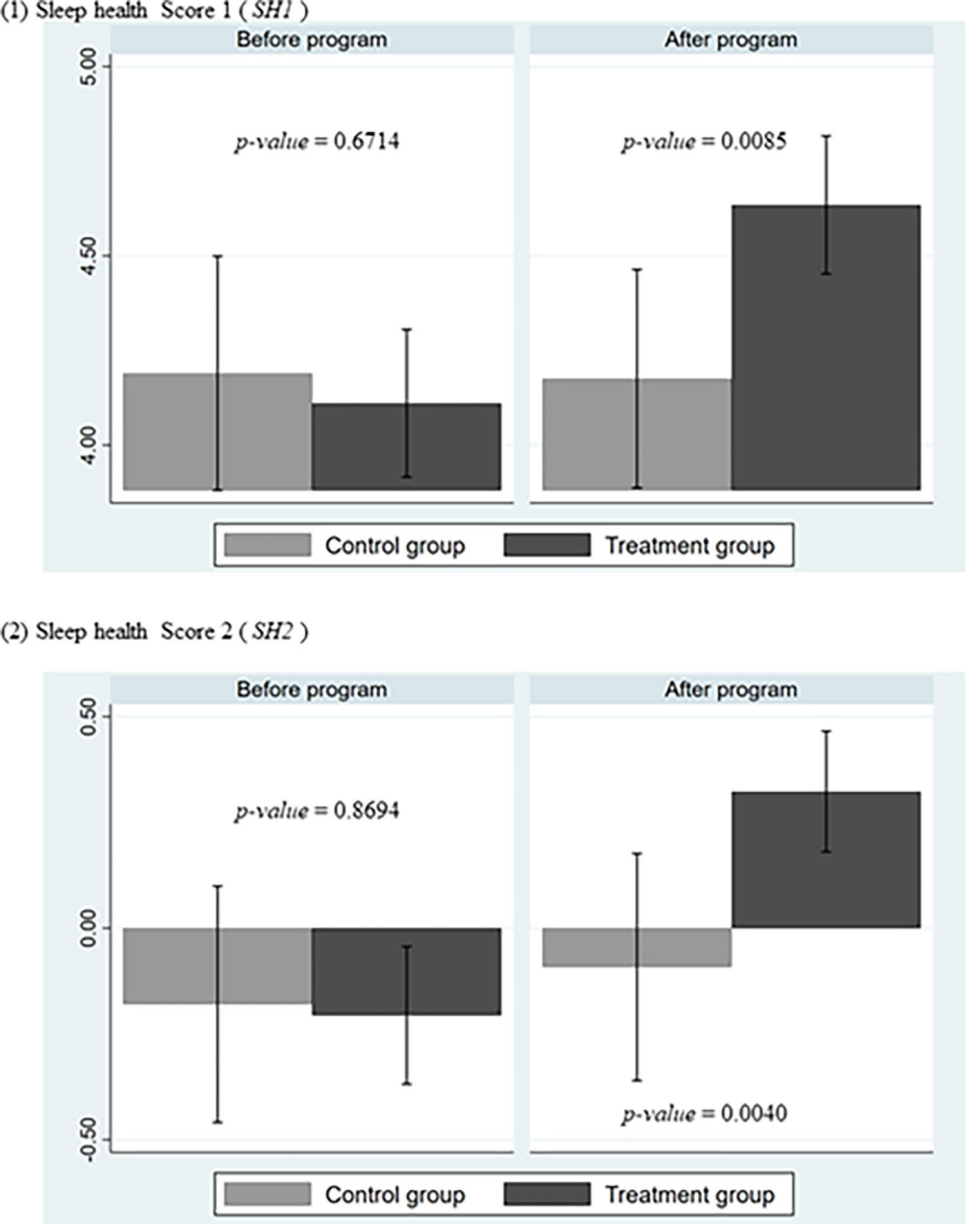
Changes before and after the intervention. (Notes) The graphs show the mean values of Sleep Health Scores 1 and 2 (SH1 and SH2) of 145 participants in the treatment group and 57 participants in the control group at each time point before and after the program. The larger the value on the vertical axis, the better the sleep health is ensured. The p values shown in the figure indicate the results of significance tests for the two groups before and after the program, indicating that although there was no statistically significant difference between the two groups before the intervention, the sleep improvement in the treatment group was statistically significant at the 1% level after the intervention.

We first present simple graphs to show the extent to which sleep health improved by comparing the pretreatment and posttreatment sleep health indices of the treatment and control groups. Fig 3 shows the mean values of (1) *SH1* and (2) *SH2* for the treatment and control groups separately for the pretreatment and posttreatment periods. The results of the t tests for the difference of means between the two groups are shown by p values in the figure. The figure shows no significant difference between the two groups for either *SH1* or *SH2* before treatment. However, we found statistically significant differences at the 1% level for both *SH1* and *SH2* after treatment, suggesting that the sleep improvement program improved sleep in the treatment group.

In the following, we rigorously examine these results by estimating the three models (ANCOVA and two 2SLS models through diligent participation and sleep improvement) explained in the previous section.

### 4–2. Treatment effects on sleep-related behavior

We now examine the effect of the program on sleep-related behavior. Using the ANCOVA model in Eq 3, we estimate the treatment effect on the behavioral changes related to sleep, listed as (a)-(i) in section 2-2-1. All variables were scored on a four-point scale, where a lower number is interpreted as less frequent.

Table 4 shows the results. Among the nine sleep-related behaviors, we found a statistically significant improvement for the treatment group in the first two items. According to column (a), participants in the treatment group were 0.42 SD less likely to “do something unrelated to sleep in bed” than those in the control group. Column (b) shows that participants in the treatment group were 0.39 SD less likely to “spend time in a well-lit room until about an hour before going to bed” than those in the control group. Since the dependent variables are categorical, we also estimated ordered logit models and confirmed that the results are qualitatively the same. We did not find any significant effects for other sleep-related behaviors (columns c—i).

**Table 4:** Effect of intervention on behavioral change in sleep.

	(a)	(b)	(c)	(d)	(e)	(f)	(g)	(h)	(i)
	do anything in bed that is not related to sleep	spend time in a brightly lit room before going to bed	sleep with the lights on	feel my hands and feet are cold before going to bed	take naps during work or breaks	have a habit of exercising on a regular basis	drink alcohol within 2 hours before going to bed	skip a meal after waking up	eat within an hour before going to bed
									
treatment	-0.378***	-0.381***	-0.0611	0.00143	0.140*	0.246	0.0485	-0.150	-0.0747
	(0.125)	(0.136)	(0.0775)	(0.140)	(0.0731)	(0.162)	(0.112)	(0.107)	(0.115)
sample sizes	202	202	202	202	202	202	202	202	202
R-squared	0.516	0.207	0.446	0.467	0.606	0.504	0.673	0.621	0.436
control variables	YES	YES	YES	YES	YES	YES	YES	YES	YES

(Notes) 1. Robust standard errors in parentheses.

2. ***, **, and * indicate statistical significance at the 1%, 5%, and 10% levels.

3. The variables described in section 2.2.4 are used as control variables in the estimation but are omitted from the table.

### 4–3. Treatment effects on sleep improvement

#### 4-3-1. Effects on sleep health indices (*SH1* and *SH2*)

Using *SH1* and *SH2* as dependent variables, we next estimated the effect of the program on sleep health improvement using Eq 3. The first column of Table 5 shows that the SH1 index rose by 0.455 points (0.38 SD) in the treatment group compared to the control group. Additionally, in the second column, the results show that the program improved the sleep health index *SH2* in the treatment group by 0.40 points (0.40 SD) compared to the control group. Although the methods of generating *SH1* and *SH2* are different, it is noteworthy that the extent of improvement in sleep health is approximately the same in terms of standard deviation. In what follows, we will focus on *SH2*.

**Table 5:** Effect of intervention on sleep improvement.

	(1)	(2)	(3)	(4)	(5)	(6)	(7)	(8)	(9)	(10)	(11)	(12)	(13)
	Sleep health score		sub-scales of SH2										
	SH1	SH2	satisfaction	daytime alertness		sleep efficiency					duration	timing	regularity
	Sleep health score 1	Sleep health score 2	the level of satisfaction with recent sleep	feel sleepy, such as yawning, drowsy, or tired within 4 hours of waking	feel drowsy at work	wake up more than an hour early and have trouble going back to sleep	have trouble sleeping through the night and wake up in the middle of the night	feel foggy and can’t get up right away even when the alarm goes off	think a lot, repeatedly turning over in bed, takes more than 30 min to fall asleep	fall asleep so fast that I lose consciousness within 5 minutes	waking time-bedtime is 6-8 hour	the midpoint is in the normal range of 2 AM to 4 AM	sleep longer than workday on your days off
treatment	0.455***	0.400***	0.289***	-0.271**	-0.0367	-0.0439	0.136	-0.366***	-0.0871	0.0480	-0.0556	0.0726	-0.196
	(0.159)	(0.122)	(0.102)	(0.129)	(0.120)	(0.0837)	(0.155)	(0.127)	(0.123)	(0.152)	(0.0600)	(0.0667)	(0.145)
changes in weekly days of WFH	0.0306	-0.00895	0.0554	0.0392	0.00991	-0.00282	0.261**	0.0643	0.0146	-0.119	0.00870	-0.0102	-0.0379
	(0.144)	(0.0934)	(0.0908)	(0.0951)	(0.0911)	(0.0574)	(0.128)	(0.0917)	(0.0930)	(0.106)	(0.0431)	(0.0471)	(0.111)
being promoted	0.101	0.0224	-0.00266	-0.682***	0.414	0.344*	-0.461	0.213	0.262	-0.120	-0.0441	-0.0828	0.743
	(0.393)	(0.303)	(0.279)	(0.255)	(0.621)	(0.190)	(0.520)	(0.235)	(0.324)	(0.398)	(0.167)	(0.284)	(0.755)
increase in workload	0.149	-0.0127	0.157	-0.0135	0.0408	0.105	0.195	0.0173	-0.0403	-0.326**	0.0410	-0.0374	-0.0239
	(0.178)	(0.125)	(0.104)	(0.137)	(0.128)	(0.104)	(0.171)	(0.142)	(0.110)	(0.149)	(0.0611)	(0.0650)	(0.151)
my boss has changed	-0.443	-0.222	-0.354	0.586*	0.294	-0.189	-0.0186	0.0367	-0.338	0.188	-0.0537	-0.0262	-0.109
	(0.391)	(0.274)	(0.231)	(0.348)	(0.276)	(0.121)	(0.312)	(0.234)	(0.314)	(0.261)	(0.131)	(0.134)	(0.371)
moved residence	0.301	0.0630	0.492*	0.0834	-0.215	-0.0104	-0.447	0.0647	-0.0542	-0.755*	-0.0172	0.0697	-0.0435
	(0.448)	(0.375)	(0.259)	(0.434)	(0.263)	(0.246)	(0.301)	(0.324)	(0.226)	(0.440)	(0.162)	(0.0733)	(0.475)
there were other stressful things	0.429**	0.287	0.131	-0.0240	-0.136	0.0680	0.0656	-0.151	-0.185	0.260	0.139	0.141*	-0.0881
	(0.196)	(0.183)	(0.151)	(0.176)	(0.206)	(0.207)	(0.287)	(0.241)	(0.162)	(0.230)	(0.0996)	(0.0839)	(0.270)
sample size	200	200	202	202	202	202	202	202	202	202	200	200	202
R-squared	0.369	0.518	0.307	0.293	0.438	0.535	0.373	0.527	0.490	0.494	0.262	0.367	0.515
control variables	YES	YES	YES	YES	YES	YES	YES	YES	YES	YES	YES	YES	YES

(Notes) 1. Robust standard errors in parentheses.

2. ***, **, and * indicate statistical significance at the 1%, 5%, and 10% levels.

3. The variables described in section 2.2.4 are used as control variables in the estimation, but only some of them are included in the table.

#### 4-3-2. Effects on SH2 subscales

To identify which subitems of SH2 improved through the intervention, we further estimate Eq 3 by using the six subscales of *SH2* as dependent variables. The results are shown in the third and subsequent columns of Table 5.

A statistically significant effect was confirmed for *sleep satisfaction*, which rose 0.289 points after the intervention. Regarding *daytime alertness*, the intervention effect was found to reduce the frequency of “feeling sleepy, such as yawning, drowsy, or tired within 4 hours of waking” by 0.27 (column 4). Regarding *sleep efficiency*, the intervention was effective in reducing the frequency of “feeling foggy and can’t get up right away even when the alarm goes off” by 0.37 (column 8). No statistically significant effects were found for *sleep duration*, *timing*, or *regularity*. These results suggest that the intervention had a positive effect on *sleep satisfaction* and some other aspects of *daytime alertness* and *sleep efficiency*, leading to an improvement in *SH2*.

There are some other notable findings in the table. First, from the results in column 7, an increase in the number of days spent working from home may have caused the participants to experience more “trouble sleeping through the night and waking up in the middle of the night.” Telework may have an adverse effect on sleep, such as nocturnal awakening, potentially due to changes in work schedule, such as working until just before going to bed or waking up later. However, no adverse effects other than nocturnal awakening were detected for telework; thus, any adverse effect it has on sleep may be limited. Second, those who had an increase in workload may have experienced a decrease in “falling asleep fast enough to lose consciousness within five minutes” (column 10). Third, for those who were promoted during the RCT implementation period, “feeling sleepy” (column 4) was statistically negative and significant, while “waking up more than an hour early and having trouble going back to sleep” (column 6) was statistically positive and significant. This result can be interpreted as suggesting that immediately after a promotion, workers are more likely to feel nervous and excited by the new tasks they are given and are more likely to have trouble sleeping. No effects were detected for items other than these two, however.

### 4–4. Treatment effects on productivity

#### 4-4-1. ATT on productivity

We next estimate Eq 3 using two productivity measures, *Productivity index 1* (questionnaire-based productivity composite index) and *Productivity index 2* (WLQ). We control for the pretreatment productivity index and other control variables explained in section 2.

Column 2 in Table 6 shows that *Treatment* has a statistically significant coefficient when using the total WLQ score (*Productivity index 2*) as the dependent variable. In other words, the sleep program resulted in a 1.45-point higher total WLQ score for the treatment group than for the control group. *Productivity index 1* and the subscales of *Productivity index 2* show some improvement but only weakly significant differences at best.

**Table 6:** Effect of intervention on productivity improvement (ANCOVA).

	(1)		(2)	(3)	(4)	(5)	(6)
	Productivity Index 1		Productivity Index 2 (WLQ)				
	a synthetic variable		total	time management	physical tasks	mental-Interpersonal Tasks	output tasks
							
treatment	0.209*		1.454**	5.360*	3.741	5.204*	6.395*
	(0.119)		(0.731)	(2.993)	(3.925)	(2.896)	(3.557)
sample sizes	201		153	158	153	158	158
R-squared	0.615		0.514	0.447	0.414	0.527	0.424
control variables	YES		YES	YES	YES	YES	YES

(Notes) 1. Robust standard errors in parentheses.

2. ***, **, and * indicate statistical significance at the 1%, 5%, and 10% levels.

3. The variables described in section 2.2.4 are used as control variables in the estimation but are omitted from the table.

However, as mentioned in the previous section, the causal effects of the intervention presented in Table 6 might have been weakened because not all participants actively used the app and its advice to improve their sleep-related behaviors. Therefore, in the following sections, we attempt to evaluate the effect of diligent participation on sleep improvement using two-stage least squares.

#### 4-4-2. Effect of diligent efforts on productivity (2SLS)

In what follows, we use the 2SLS method to determine whether diligent participants in the sleep program improved their productivity. This estimate can be interpreted as the local average treatment effect (LATE) of diligent participation as expressed by the model, Eqs 4 and 5, in section 3–2.

The results are shown in Table 7. The endogenous variable, *diligent participation*, has a statistically significant coefficient when the dependent variable is the total score (*Productivity index 2*), the time management score, or the work performance score of the WLQ (columns 2, 3, and 6). In other words, when the assignment to the treatment group caused participants to engage in the program diligently, their total score, time management score, and output tasks score on the WLQ improved by 2.62 points, 9.31 points, and 11.36 points on average, respectively. However, no significant effects on physical task scores, mental-interpersonal task scores, or *Productivity index 1* (productivity composite measures) were found at the 5% level.

**Table 7:** Effect of diligent participation on productivity (LATE).

	(1)		(2)	(3)	(4)	(5)	(6)
	Productivity Index 1		Productivity Index 2 (WLQ)				
	a synthetic variable		total	time management	physical tasks	mental-Interpersonal Tasks	output tasks
diligent participation	0.383*		2.622**	9.314**	6.816	9.363*	11.36**
	(0.215)		(1.196)	(4.629)	(6.275)	(4.798)	(5.774)
sample sizes	199		151	156	151	156	156
R-squared	0.622		0.482	0.447	0.429	0.523	0.370
control variables	YES		YES	YES	YES	YES	YES

(Notes) 1. Robust standard errors in parentheses.

2. ***, **, and * indicate statistical significance at the 1%, 5%, and 10% levels.

3. The variables described in section 2.2.4 are used as control variables in the estimation but are omitted from the table.

4. Regarding *Productivity Index 2*, the sample varies by subscale.

#### 4-4-3. Effect of sleep improvement on productivity (2SLS)

We next estimated the effect of the intervention on productivity through improvements in the sleep health indices *SH1* and *SH2*. A 2SLS estimation with the posttreatment *SH2* as the endogenous variable and the treatment group dummy as the instrument variable was conducted using Eqs 6 and 7 in section 3–2. Table 8 confirms significant causal effects of improved sleep health (*SH2*) on *Productivity index 1* and *Productivity index 2*. The latter includes the WLQ’s total score and its subscales: time management, mental interpersonal tasks, and output tasks. Among participants in the treatment group, a one standard deviation improvement in the *SH2* led to a 0.59-point increase in *Productivity index 1* (column 1), a 3.00 point increase in the WLQ total score (column 2), a 10.94 point increase in the time management score (column 3), an 11.37 point increase in the mental interpersonal tasks score (column 5), and a 12.11 point increase in the output tasks score (column 6). We reran the same model estimation using each of the *SH2* subscales as an endogenous variable to examine which dimension of the sleep health measure mediated the treatment effect on productivity. Statistically significant effects were found when using *satisfaction* and *daytime alertness* as the endogenous variables. To summarize, productivity increased through the improvement of *SH2*, which was primarily mediated by *satisfaction* and *daytime alertness* among six sleep health dimensions.

**Table 8:** Effect of intervention on productivity through improved sleep (2SLS).

	(1)		(2)	(3)	(4)	(5)	(6)
	Productivity Index 1		Productivity Index 2 (WLQ)				
	a synthetic variable		total	time management	physical tasks	mental-Interpersonal Tasks	output tasks
							
sleep improvement	0.594**		3.004**	10.94**	7.784	11.37**	12.11**
	(0.294)		(1.263)	(5.075)	(6.800)	(4.766)	(5.882)
sample sizes	199		152	157	152	157	157
R-squared	0.600		0.482	0.416	0.383	0.540	0.398
control variables	YES		YES	YES	YES	YES	YES

(Notes) 1. Robust standard errors in parentheses.

2. ***, **, and * indicate statistical significance at the 1%, 5%, and 10% levels.

3. The variables described in section 2.2.4 are used as control variables in the estimation but are omitted from the table.

4. Regarding *Productivity Index 2*, the sample varies by subscale.

Note that the purpose of this paper is to examine the relationship between sleep improvement and productivity in the general workforce, rather than in patients with insomnia. Therefore, we did not set any special criteria for recruiting subjects within the company but recruited a wide range of workers who were interested in improving their sleep health. However, we must be aware of the possibility that some of the applicants may have had insomnia or depression, which could substantially affect the effectiveness of the sleep hygiene program. To prevent such cases from affecting our results significantly, we also ran the same regression analysis with a subsample, excluding observations based on several of the criteria (those who had mentioned “sleepless,” “need improvement in physical condition,” or “Feeling depressed almost every day”) as a robustness check. We obtained similar results to those reported in this paper even after excluding those observations.

### 4–5. Economic return to sleep improvement programs

In section 4–4, we measured the effects on productivity through three pathways: average effects on the treatment group, local average treatment effects of diligent participants, and indirect effects through sleep improvement. Based on those estimators, we evaluated the economic return to sleep improvement programs using the total WLQ score. Although we should look at the net return on investment, the total cost of the sleep improvement program was not disclosed to us by the firm, so we only evaluated the economic benefit.

The following assumptions are made in our calculation.

1. The range of change in the overall WLQ score equals the rate of change in productivity.
2. The average productivity per person per year is 8 million yen (approximately 60,000 US dollars).
3. For the 112 participants in the treatment group who responded to the WLQ, the estimated effect of the intervention continued for one year.

The first assumption is based on our understanding that the WLQ was originally designed to measure the value of productivity loss (Lerner et al. [37]), and the manager in charge of the program at the firm said that he adopted the WLQ survey because he felt that the loss rate of the WLQ was closest to his actual perception of productivity loss. Note that the calculation of the cost of presenteeism using WLQ has been shown in many previous studies. See, for example, Mitchell and Bates [41], who calculated the loss due to presenteeism based on the WLQ after matching people with and without the disease using propensity scores. For assumption 2, we set the average productivity at 8 million yen because the average labor cost of listed manufacturing companies is said to be approximately 8 million yen.

According to Table 6, the effect on the treatment group (ANCOVA) has a coefficient of 1.454, which can be interpreted as an improvement of 1.454 percentage points in the loss of value of WLQ. Our treatment sample contains the total index of WLQ for 112 people, for whom the economic benefit is 0.01454 * 8 million yen * 112 people = 13.03 million yen (97,700 US dollars).

Next, the local average treatment effect (LATE) of diligent participation in Table 7 is 2.622. Although it is not shown in the table, the ratio of participants in the treatment group who made a serious effort to comply was 55.5%. Therefore, the improvement in value through diligent participation is 0.555 * 0.02622 * 8 million yen * 112 participants = 13.04 million yen (97,800 US dollars).

Finally, the coefficient of sleep improvement (i.e., endogenous variable in 2SLS) in Table 8 is 3.00, but the impact of the intervention on the sleep health index *SH2* is known to be 0.503 from the first-stage estimates (although not shown in the table). Hence, the economic benefit of improved sleep is 0.503 * 0.03004 * 8 million yen * 112 people = 13.53 million yen (101,500 US dollars). In summary, the economic benefits of all three estimation methods are almost the same, suggesting that the increase in productivity is due to the improved sleep of those who took the intervention seriously (55.5% of all participants), and the other possible pathways were largely negligible.

The most uncertain of our assumptions is the third assumption, the duration of the intervention effect. The Hawthorne effect may have exaggerated the short-term effect of the program, and the improved sleep-related behaviors and productivity gains may be reversed in a few months. Conversely, there is also a possibility that participants who realized the importance of sleep may continue to take steps to improve their sleep, and the effects may last for more than a year.

### 4–6. Who improved and who did not?

As mentioned in section 4–3, not all participants in the treatment group improved their sleep health through the sleep improvement program. According to previous studies, the effects and dropouts of RCTs vary according to individual characteristics, personality traits and level of education (see, for example, Bagby et al. [42], Edmonds et al. [43], Schmidt et al. [44]). Given these findings, in this last section, we investigate whether sleep health improvement is more pronounced for individuals with specific attributes. In sections 4–3 and 4–4, most analyses were limited to the participants who responded to the WLQ. In this section, we focus on the 145 participants in the treatment group for whom follow-up survey data are available.

We use two dependent variables that specify those whose sleep has improved. The first is a binary variable in which one is assigned to those whose *sleep satisfaction* increased by at least one point on a four-point scale and zero otherwise. The second is a binary variable that takes one if the change in the sleep health score *SH2* after the treatment corresponds to the top 40th percentile and zero otherwise. These variables are regressed on explanatory variables in the logit model. For the covariates, we employ responses to the following questions in the baseline survey: (question 1) “Please answer frankly about your current state of mind when starting the program” and (question 2) “If you have made health-related habitual efforts (exercise, diet, etc.) in the past, please select an answer that most closely matches your own tendency. The options for question 1 are “I don’t like to start new things”, “I am interested in starting new things, but I don’t want to change my life too much”, “I want to challenge new things as much as I can”, and “I want to challenge new things aggressively even if it is a little difficult”. Participants are asked to choose one of the four options for questions 1 and 2. We create a dummy variable that is assigned one for those who chose the last option, “Challenge aggressively”, which corresponds to the highest willingness for question 1, and zero for the others, as a proxy variable for the person’s willingness to make a serious effort. For question (2), the options are “I get bored in the middle and don’t continue to the end,” “I can continue if I have the right support,” “I can continue to work hard alone to achieve my goal,” and “I feel a sense of accomplishment in completing the task and can go on and on by myself.” We generate a dummy that takes one for those who chose “a sense of accomplishment in completing the task (highest perseverance)” and zero for the others as a proxy for the person’s perseverance. In addition to basic attributes such as gender, age, and family structure, major changes in work and living conditions during the program period and sleep conditions before the program started are added as explanatory variables.

The estimation results are presented in Table 9. The variables of *willingness* and *perseverance* are shown to have a significantly positive effect on *sleep satisfaction* (columns 1 to 3), while both are less significantly associated with *SH2* (columns 4 to 6). The analysis also revealed that the probability of sleep improvement was higher for those with lower prior sleep satisfaction (columns 1 to 3) or sleep health conditions (columns 4 to 6). In addition, we find that it is difficult to improve *sleep satisfaction* for those in their 40s and older.

**Table 9:** Relationship between sleep health improvement and individual attributes.

	(1)	(2)	(3)	(4)	(5)	(6)
	improvement in sleep satisfaction			improvement of SH2		
state of mind at the start:	2.105***		2.365***	1.055*		1.124*
"challenge aggressively"=1	(0.706)		(0.805)	(0.540)		(0.634)
health-related habitual efforts:		2.733***	2.746***		0.617	0.357
"feel a sense of accomplishment"=1		(0.745)	(1.031)		(0.618)	(0.808)
sleep satisfaction (before program)	-3.510***	-3.475***	-4.348***			
	(0.680)	(0.782)	(0.931)			
SH2 (before program)				-1.379***	-1.352***	-1.408***
				(0.295)	(0.291)	(0.293)
female (base=male)	0.505	0.774	1.251**	-0.437	-0.437	-0.254
	(0.595)	(0.585)	(0.638)	(0.544)	(0.565)	(0.596)
age (base=20s) : 30s	-1.082	-1.183*	-1.083	-0.485	-0.599	-0.417
	(0.659)	(0.711)	(0.772)	(0.701)	(0.729)	(0.731)
40s	-2.341***	-2.181**	-2.804***	-0.0431	-0.0888	-0.203
	(0.850)	(0.885)	(0.924)	(0.769)	(0.789)	(0.816)
over 50	-2.142**	-1.983**	-2.749***	-0.790	-0.820	-0.708
	(0.861)	(0.806)	(0.945)	(0.721)	(0.732)	(0.730)
family members (base=single): infants and/or children	-0.361	-0.132	0.142	0.281	0.382	0.263
	(0.720)	(0.718)	(0.816)	(0.519)	(0.534)	(0.555)
spouse or partner	0.548	0.408	0.808	0.570	0.527	0.494
	(0.694)	(0.713)	(0.821)	(0.607)	(0.620)	(0.630)
others (sibilings, grandparents, roommates)	0.295	0.290	0.868	0.775	0.696	0.886
	(0.861)	(0.897)	(1.001)	(1.297)	(1.260)	(1.379)
workplace support: change in supervisor’s support	0.0734	0.200	0.349	-0.465	-0.378	-0.411
	(0.301)	(0.284)	(0.338)	(0.389)	(0.405)	(0.389)
change in colleagues’ support	0.275	0.257	0.0796	0.511	0.463	0.456
	(0.326)	(0.340)	(0.358)	(0.332)	(0.341)	(0.348)
changes in days of WFH	-0.393	-0.745*	-0.702	-0.270	-0.396	-0.368
	(0.397)	(0.405)	(0.459)	(0.373)	(0.382)	(0.386)
changes in degree of job control	-0.0437	-0.0605	-0.0568	0.137	0.119	0.137
(can set my own pace)	(0.416)	(0.337)	(0.517)	(0.346)	(0.305)	(0.342)
changes in degree of job control	-0.774*	-0.539	-0.553	0.0208	0.0957	0.0718
(can decide order and way of my work)	(0.442)	(0.389)	(0.472)	(0.353)	(0.351)	(0.370)
sample sizes	136	136	132	140	140	136
Pseudo R-square	0.398	0.389	0.452	0.289	0.275	0.294
control variables	YES	YES	YES	YES	YES	YES

(Notes) 1. Robust standard errors in parentheses.

2. ***, **, and * indicate statistical significance at the 1%, 5%, and 10% levels.

3. The purpose at the start of the program (multiple responses) and major changes in work and life during the program period are used as control variables but are omitted from the table.

## 5. Discussion and conclusion

Using a randomized controlled trial of a sleep improvement program in a firm’s workforce, this paper examined the extent to which improved sleep health increases work productivity. The results of the analysis revealed the following. First, participants in the sleep improvement program showed a statistically significant improvement in sleep health compared to the control group after accounting for a variety of work and lifestyle factors that may affect sleep. Second, the treatment group also exhibited a statistically significant improvement in work productivity, which was fully explained by the effect through sleep improvement. The total effect sizes for productivity improvement were similar whether the ANCOVA or 2SLS estimation methods that capture the effect through diligent participation or sleep improvement were used. Although it is possible for anyone to experience worsening sleep due to various changes and events in their work and personal lives, promoting sleep health through the use of information technology such as sensing devices may help improve sleep deficiency and restore productivity. However, the results of this paper also reveal that the effect is heterogeneous depending on individual characteristics such as age and willingness and perseverance to improve sleep health. When implementing proactive corporate health interventions, it is important to plan ahead, identifying groups of employees who might benefit most from interventions and incorporating additional nudges to support those who have difficulty changing their behavior.

Currently, many developed countries are experiencing an aging population. Japan has the oldest population in the world, with the proportion of people aged 65 and over recorded at 28.7% of the total population in 2020, and this proportion is projected to rise to 35.3% by 2040. To cope with the aging of the population, the government is requiring companies to try to retain their employees until the age of 70 under the revision Act on Stabilization of Employment of Elderly Persons from April 2021. There is concern that as the number of people with sleep problems increases with age (see, for example, Léger et al. [45], Dregan and Armstrong, [46, 47], van de Straat and Bracke [5]), their productivity will also be affected. According to the *National Survey on Basic Living Conditions* (Ministry of Health, Labour and Welfare 2019), the proportion of people who do not get enough rest from sleep, as mentioned at the beginning of this paper, is reported to increase with age. In addition, many Japanese people sleep less (Walch et al. [48]), and estimates based on the *Survey on Time Use and Leisure Activities* show that sleep duration among full-time workers has been on a downward trend for the past 30 years (Kuroda [49]). A similar downward trend of sleep duration in the United States was also reported by Sheehan [50] (for an international comparison of sleep duration, see Whinnery et al. [51], Kuula et al. [52]). In addition to the aging of the population, we cannot ignore the impact of the global shift to a “24-hour economy” and the accompanying extension of working hours due to the spread of smartphones and telecommuting. We now face blurred boundaries between life and work, leading to a deterioration in sleep regularity and an increase in the number of people with sleep deficiency.

As this paper has shown, poor sleep health leads to a decline in productivity, which is a major loss for the economy. The problem, however, is that when a person experiences chronically poor sleep, he or she may feel it is normal, and it becomes difficult for the person to recognize the decline in productivity. Additionally, even if the person is aware of productivity loss, companies may not be able to detect presenteeism. A person can see a doctor if he or she has extreme insomnia, but few people will see a doctor for a slight loss of productivity if it does not interfere with their daily lives. As a result, people tend to neglect their daily sleep, and even if they want to get a good night’s sleep, they are unlikely to actively gather information to improve their sleep health or examine related bad habits that negatively affect sleep. Companies should understand that the importance of sleep health is not easily recognized by individuals and may consider implementing proactive health management, such as providing sleep improvement measures or subsidizing the cost of sleep technology for employees.

Finally, we discuss the limitations of our paper. First, we conducted an RCT on white-collar day workers as the main target group, and productivity improvements through sleep improvement were confirmed for those workers. However, it is not clear whether similar improvements can be expected for shift workers and blue-collar workers in various industries, such as construction, manufacturing, and transportation. It is necessary to verify the results by expanding the target occupations. Second, this paper targets workers who are interested in improving their sleep, and the sample size is not large (approximately 200 workers). It is necessary to examine the results of a larger sample including those who have sleep problems but are not interested in the sleep health measures or those who are strongly resistant to the interventions. Third, the effects of the RCT analyzed in this paper were based on data immediately after the program was implemented, and it is unclear how long the changes in the participants’ behavior to improve their sleep might last. Even if the advice given by the sleep app was temporarily beneficial, the improved behaviors may be reversed as soon as individuals stop using the app or sleep measurement device. Fourth, it is also necessary to examine whether productivity gains due to improved sleep are a temporary phenomenon or whether they persist. It will be important to monitor changes over time by conducting multiple follow-up surveys of RCT subjects. Fifth, the usage of actigraphy data recorded by the sleep device in the treatment group is another future challenge. We created the third sleep health score (*SH3*) using the daily sleep data recorded for the treatment group to examine the impact on presenteeism. We conducted the analysis limiting samples only to the treatment group by accounting for possible selection biases (note that the actigraphy data were only available for the treatment group). There was no statistically significant effect on either *productivity index 1* or *2* through the improvement of *SH3*. This is not necessarily due to limited variation in sleep health within the treatment group because when a similar analysis was conducted for *SH2* using only treatment group samples, *productivity index 1* was significantly associated with the improvement in *SH2*. There is a possibility that actigraphy sleep data do not sufficiently capture the factors that affect sleep satisfaction. According to Tal et al. [35], the device had a sensitivity of 92.5% in determining sleep/awake state. When breaking sleep into rapid eye movement (REM), light sleep and slow wave sleep, those sensitivities dropped to 53.7, 64.9, and 56.2, respectively. In the future, it may be necessary to explore more effective ways to utilize actigraphy sleep data, such as the use of machine learning. These points remain to be addressed.

## Supporting information

S1 File (PDF)
