# Correction: The impact of a mobile app-based corporate sleep health improvement program on productivity: Validation through a randomized controlled trial

**DOI:** 10.1371/journal.pone.0295158

**Published:** 2023-11-28

**Authors:** The PLOS ONE Staff

The publisher apologizes for the errors. There is an error in affiliations 2 and 3 for authors, Sachiko Kuroda and Hideo Owan. The correct affiliation 2 is: Faculty of Education and Integrated Arts and Sciences, Waseda University, Japan; and the correct affiliation 3 is: The Research Institute of Economy, Trade and Industry, Tokyo, Japan.

In the Analysis of covariance (ANCOVA) subsection of the Estimation strategy, there is an error in the first equation. The correct Eq 1 is:

$${\hat{\gamma }}_{ancova}=({\bar{Y}}_{POST}^{T}-{\bar{Y}}_{POST}^{C})-\hat{\theta }({\bar{Y}}_{PRE}^{T}-{\bar{Y}}_{PRE}^{C}).$$ (1)

where ${\bar{Y}}_{POST}^{T}=\frac{1}{n_{T}}\sum_{i\in T}Y_{i1},\;{\bar{Y}}_{POST}^{C}=\frac{1}{n_{C}}\sum_{i\in C}Y_{i1},\;{\bar{Y}}_{PRE}^{T}=\frac{1}{n_{T}}\sum_{i\in T}Y_{i0}$, and ${\bar{Y}}_{PRE}^{C}=\frac{1}{n_{C}}\sum_{i\in C}Y_{i0}.\;Y_{i,t}$ are the outcome measures for worker *i* from the baseline (t = 0) and follow-up (t = 1) surveys, and *n*_*T*_ and *n*_*C*_ are the number of workers in the treatment group and in the control group, respectively.

## References

[1] Kawata Y, Kuroda S, Owan H (2023) The impact of a mobile app-based corporate sleep health improvement program on productivity: Validation through a randomized controlled trial. PLoS ONE 18(10): e0287051. doi: 10.1371/journal.pone.0287051 37796855 PMC10553342

