# Supplementary material for: The impact of a mobile app-based corporate sleep health improvement program on productivity: Validation through a randomized controlled trial
Source: PLoS One. 2023 Oct 5;18(10):e0287051. doi: 10.1371/journal.pone.0287051 (PMC10553342; doi:10.1371/journal.pone.0287051)
Supplement: S1 File — (PDF) [file pone.0287051.s001.pdf]

S1 Table: Basic statistics (other than Table 3)

	Full sample			Treatment group			Control group		
	(1) sample sizes	(2) mean	(3) standard deviation	(4) sample sizes	(5) mean	(6) standard deviation	(7) sample sizes	(8) mean	(9) standard deviation
**Behavioral Changes in Sleep**									
**"Do you ever . . . " (the frequency in the last week or two was asked)**									
(a) do anything in bed that is not related to sleep	404	3.11	1.11	290	2.99	1.16	114	3.41	0.94
(b) spend time in a brightly lit room before going to bed	404	3.69	0.73	290	3.63	0.78	114	3.82	0.54
(c) sleep with the lights on	404	1.20	0.55	290	1.18	0.51	114	1.26	0.64
(d) feel my hands and feet are cold before going to bed	404	1.83	1.03	290	1.81	1.03	114	1.89	1.05
(e) take naps during work or breaks	404	1.33	0.69	290	1.34	0.68	114	1.31	0.72
(f) have a habit of exercising on a regular basis	404	2.10	1.00	290	2.17	0.97	114	1.92	1.07
(g) drink alcohol within 2 hours before going to bed	404	1.86	1.02	290	1.88	1.00	114	1.82	1.08
(h) skip a meal after waking up	404	1.69	1.05	290	1.65	1.04	114	1.78	1.10
(i) eat within an hour before going to bed	404	1.62	0.78	290	1.57	0.76	114	1.73	0.82
**Productivity Index 1 (the frequency in the last two weeks on a 10-point Likert scale)**									
(a) I feel fit with no physical discomfort or major fatigue.	403	5.32	2.16	290	5.43	2.22	113	5.03	1.98
(b) I can get to work quickly with a clear head first thing in the morning.	403	5.67	2.16	290	5.83	2.16	113	5.27	2.12
(c) I am able to organize my thoughts.	403	6.00	1.88	290	6.13	1.86	113	5.65	1.89
(d) I feel I have light footwork and am able to communicate well with others.	403	6.41	1.93	290	6.51	1.92	113	6.16	1.93
(e) I am not irritated by trivial things, and I feel comfortable.	403	5.69	2.14	290	5.72	2.11	113	5.63	2.21
(f) I can re-focus on work after being distracted.	403	6.09	1.76	290	6.21	1.71	113	5.79	1.85
(g) I am very dedicated to my work and other things.	403	6.34	1.89	290	6.52	1.86	113	5.88	1.90
(h) I can carry out my tasks accurately and carefully.	403	6.31	1.78	290	6.50	1.73	113	5.82	1.84
(i) I have a broad perspective and am able to work ahead of time.	403	6.09	1.80	290	6.27	1.71	113	5.61	1.93
(j) I can finish work on time or ahead of schedule and not at the last minute.	403	5.32	2.19	290	5.57	2.14	113	4.67	2.18
(k) I am able to tackle things with perseverance.	403	6.38	1.82	290	6.60	1.75	113	5.82	1.90
(l) I am able to concentrate while working.	403	5.73	2.16	290	5.88	2.13	113	5.34	2.19
(m) I can think quickly when making decisions.	403	5.65	1.89	290	5.84	1.85	113	5.13	1.92
(n) I get ideas and inspiration.	403	5.68	1.82	290	5.84	1.83	113	5.25	1.75
(o) I am able to think about things in depth.	403	5.93	1.83	290	6.12	1.82	113	5.46	1.78
composite index	403	-0.03	0.99	290	0.07	1.01	113	-0.29	0.89
**Productivity Index 2**									
time manegement	358	82.04	16.69	259	82.84	17.08	99	79.95	15.52
physical tasks	350	72.15	24.08	255	72.49	24.94	95	71.24	21.69
mental-Interpersonal tasks	358	79.90	15.36	259	81.35	15.35	99	76.09	14.79
output tasks	357	78.36	18.14	258	80.05	17.83	99	73.94	18.28
total	349	94.19	3.90	254	94.50	3.94	95	93.38	3.71

	Full sample			Treatment group			Control group		
	(1) sample sizes	(2) mean or n	(3) standard deviation or %	(4) sample sizes	(5) mean or %	(6) standard deviation or %	(7) sample sizes	(8) mean or n	(9) standard deviation or %
**Control variables**									
checking Emails two hours before going to bed	404	2.38	1.17	290	2.40	1.18	114	2.35	1.14
frequency of feeling depressed	404	2.19	0.96	290	2.18	0.98	114	2.21	0.94
workplace support: supervisor's support	404	2.65	0.96	290	2.66	0.98	114	2.63	0.92
workplace support: colleague's support	404	2.78	0.92	290	2.82	0.93	114	2.66	0.89
frequency of working from home (WFH)	404	2.75	0.95	290	2.76	0.93	114	2.73	1.00
job demands "too heavy of a workload that I am not able to handle my work in time"	404	2.62	1.00	290	2.60	0.97	114	2.68	1.05
job demand "my job requires a high level of knowledge and skill"	404	2.61	0.82	290	2.63	0.81	114	2.54	0.83
job control "I can work at my own pace"	404	2.71	0.87	290	2.72	0.87	114	2.69	0.87
job control "I can decide the order of work and how to do it myself"	404	2.94	0.74	290	2.97	0.72	114	2.88	0.79
task "In your daily work, how often are you faced with complex problems that require more than 30 minutes to come up with a solution?"	404	3.73	0.84	290	3.75	0.88	114	3.68	0.76
task "How often do you usually work as a supervisor for other employees in a day?"	404	1.70	1.01	290	1.66	1.01	114	1.81	1.00
task "How often do you perform repetitive tasks in a day that can be completed without coordination or consultation with others?"	404	2.33	0.96	290	2.32	0.94	114	2.33	1.02
task "How often do you talk with people inside and outside the company (i. e. customers, business partners, other departments) for the purpose of coordination, consultation, negotiation, and so on?"	404	3.20	0.94	290	3.20	0.96	114	3.21	0.90
task "How often do you spend time using your body, such as standing, walking, or working in a factory?"	404	1.92	1.11	290	1.88	1.03	114	2.02	1.28
shift dummy	202	1	0.5%	145	1	0.7%	57	0	0.0%
**Any experience during the program periods**									
being promoted	202	5	2.5%	145	2	1.4%	57	3	5.3%
being transferred to another department	202	0	0.0%	145	0	0.0%	57	0	0.0%
workload has increased	202	68	33.7%	145	42	29.0%	57	26	45.6%
workload has decreased	202	0	0.0%	145	0	0.0%	57	0	0.0%
supervisor has changed	202	10	5.0%	145	6	4.1%	57	4	7.0%
got married	202	2	1.0%	145	1	0.7%	57	1	1.8%
got divorced	202	0	0.0%	145	0	0.0%	57	0	0.0%
a child was born	202	0	0.0%	145	0	0.0%	57	0	0.0%
moving residence	202	6	3.0%	145	4	2.8%	57	2	3.5%
illness	202	2	1.0%	145	2	1.4%	57	0	0.0%
started nursing care of parents or other family members	202	2	1.0%	145	1	0.7%	57	1	1.8%
experienced a bereavement for a family or close friend	202	2	1.0%	145	2	1.4%	57	0	0.0%
experienced other stressful events	202	18	8.9%	145	12	8.3%	57	6	10.5%
nothing happened during the program period	202	117	57.9%	145	92	63.4%	57	25	43.9%

S1 Appendix Contactless sensing device

During the first seven days, the app collected four basic sleep-related measures, including sleep regularity, sleep duration, sleep latency, and sleep depth. In the second week, participants were advised to go to bed and wake up at the same time as much as possible, followed by a stage in which they were encouraged to attain a proper sleep duration. The app also provided weekly advice based on the individual's sleep status to ensure that the time to fall asleep was 15 minutes or less and that deep sleep was sustained for at least 72 minutes per night. Specific advice is based on the Sleep Hygiene Manual [1]. Given advice includes, for example, "get out of bed as soon as possible after waking up," "stretch before bedtime," "avoid caffeine in the evening and later," "switch your smartphone to night mode," "don't look at your smartphone when you get into bed," and "avoid drinking alcohol before bedtime". Each week, participants were presented with a menu of 37 recommendations. Participants chose a menu for the week from among the recommendations, and at the end of each day, the app checked whether the participant followed the advice, which is designed to encourage daily behavioral change.

S2 Appendix Control variables

In addition to personal attributes such as age, gender, and family structure, we control for information obtained from baseline and follow-up surveys related to work and the workplace (availability of support from superiors and colleagues, volume of work, degrees of job discretion and job control, description of tasks, shift work dummy). One of the 157 employees in the treatment group was a shift worker, but the remaining 156 employees were all day workers. We conducted the analysis excluding the shift worker, but the results did not differ from those reported in the paper. For the support of supervisors and colleagues and the degrees of job discretion and job control, we employed questionnaires based on the job demand-resource model ([2]; see S1 Table for specific questions). The information on tasks is based on the responses to the questionnaire on tasks in accordance with [3]; variables indicating the degree of abstract, routine, and manual tasks are used to account for each employee's job content.

In addition, we also control for major personal and professional events that might affect sleep during the three-month program period. Specifically, we asked employees whether they experienced any of the following during the period: promotion, transfer to another department, increased or decreased workload (e.g., new or delegated duties, increase or decrease in subordinates or junior staff to be supervised), change of supervisor, marriage, divorce, serious injury, relocation (e.g., moving residence, returning to parents' home, leaving parents' home), illness (e.g., surgery or long-term healing required), birth of a child, start of nursing care for parents or other family members, bereavement of a family member or close friend, or other stressful events (e.g., harassment or involvement in an incident). Any influence of such events on sleep and productivity will be

captured by the event dummies included as controls.

During the RCT program period, from January 8, 2021, to January 31, 2021, a state of emergency was declared in several areas, including Tokyo and Osaka. However, it is also notable that the firm recommended remote work throughout the program period, even before the emergency declaration was issued. Therefore, in the following analysis, we control for "the number of days worked at home per week in the last two weeks," which was included in the baseline and follow-up surveys.

References

[1] National Center of Neurology and Psychiatry. Support Manual for Sleep Disorders and Sleep Problems - For Public Health Nurses and Personal Assistance Professionals (in Japanese). National Center of Neurology and Psychiatry; 2016.

[2] Bakker AB, Demerouti E. Job Demands-Resources Theory: Taking Stock and Looking Forward. JOURNAL OF OCCUPATIONAL HEALTH PSYCHOLOGY. 2017 JUL;22(3):273-85.

[3] Autor DH, Handel MJ. Putting Tasks to the Test: Human Capital, Job Tasks, and Wages. Journal of Labor Economics. 2013;31(2):S59-96.
